# Supplementary material for: Crawling and Gliding: A Computational Model for Shape-Driven Cell Migration
Source: PLoS Comput Biol. 2015 Oct 21;11(10):e1004280. doi: 10.1371/journal.pcbi.1004280 (PMC4619082; doi:10.1371/journal.pcbi.1004280)
Supplement: S1 Code — (ZIP) [file pcbi.1004280.s012.zip › release/tst/doc/html/conrec_8h_source.html]

Tissue Simulation Toolkit: conrec.h Source File


Tissue Simulation Toolkit
 0.1.4.1


- Main Page
- Namespaces
- Classes
- Files

- File List
- File Members

conrec.h

Go to the documentation of this file.

1 /\*

2

3 Copyright 1996-2006 Roeland Merks

4

5 This file is part of Tissue Simulation Toolkit.

6

7 Tissue Simulation Toolkit is free software; you can redistribute

8 it and/or modify it under the terms of the GNU General Public

9 License as published by the Free Software Foundation; either

10 version 2 of the License, or (at your option) any later version.

11

12 Tissue Simulation Toolkit is distributed in the hope that it will

13 be useful, but WITHOUT ANY WARRANTY; without even the implied

14 warranty of MERCHANTABILITY or FITNESS FOR A PARTICULAR PURPOSE.

15 See the GNU General Public License for more details.

16

17 You should have received a copy of the GNU General Public License

18 along with Tissue Simulation Toolkit; if not, write to the Free

19 Software Foundation, Inc., 51 Franklin St, Fifth Floor, Boston, MA

20 02110-1301 USA

21

22 \*/

23 #ifndef \_CONREC\_H\_

24 #define \_CONREC\_H\_

25 /\*

26 Copyright (c) 1996-1997 Nicholas Yue

27

28 This software is copyrighted by Nicholas Yue. This code is base on the work of

29 Paul D. Bourke CONREC.F routine

30

31 The authors hereby grant permission to use, copy, and distribute this

32 software and its documentation for any purpose, provided that existing

33 copyright notices are retained in all copies and that this notice is included

34 verbatim in any distributions. Additionally, the authors grant permission to

35 modify this software and its documentation for any purpose, provided that

36 such modifications are not distributed without the explicit consent of the

37 authors and that existing copyright notices are retained in all copies. Some

38 of the algorithms implemented by this software are patented, observe all

39 applicable patent law.

40

41 IN NO EVENT SHALL THE AUTHORS OR DISTRIBUTORS BE LIABLE TO ANY PARTY FOR

42 DIRECT, INDIRECT, SPECIAL, INCIDENTAL, OR CONSEQUENTIAL DAMAGES ARISING OUT

43 OF THE USE OF THIS SOFTWARE, ITS DOCUMENTATION, OR ANY DERIVATIVES THEREOF,

44 EVEN IF THE AUTHORS HAVE BEEN ADVISED OF THE POSSIBILITY OF SUCH DAMAGE.

45

46 THE AUTHORS AND DISTRIBUTORS SPECIFICALLY DISCLAIM ANY WARRANTIES, INCLUDING,

47 BUT NOT LIMITED TO, THE IMPLIED WARRANTIES OF MERCHANTABILITY, FITNESS FOR A

48 PARTICULAR PURPOSE, AND NON-INFRINGEMENT. THIS SOFTWARE IS PROVIDED ON AN

49 "AS IS" BASIS, AND THE AUTHORS AND DISTRIBUTORS HAVE NO OBLIGATION TO PROVIDE

50 MAINTENANCE, SUPPORT, UPDATES, ENHANCEMENTS, OR MODIFICATIONS.

51 \*/

52

53 //=============================================================================

54 //

55 // CONREC is a contouring subroutine for rectangularily spaced data.

56 //

57 // It emits calls to a line drawing subroutine supplied by the user

58 // which draws a contour map corresponding to real\*4data on a randomly

59 // spaced rectangular grid. The coordinates emitted are in the same

60 // units given in the x() and y() arrays.

61 //

62 // Any number of contour levels may be specified but they must be

63 // in order of increasing value.

64 //

65 // As this code is ported from FORTRAN-77, please be very careful of the

66 // various indices like ilb,iub,jlb and jub, remeber that C/C++ indices

67 // starts from zero (0)

68 //

69 //=============================================================================

70

71 class Graphics;

72

73

78 int conrec(double \*\*d,

79  int ilb,

80  int iub,

81  int jlb,

82  int jub,

83  double \*x,

84  double \*y,

85  int nc,

86  double \*z,

87  Graphics \*g,

88  int colour=1);

89 // d ! matrix of data to contour

90 // ilb,iub,jlb,jub ! index bounds of data matrix

91 // x ! data matrix column coordinates

92 // y ! data matrix row coordinates

93 // nc ! number of contour levels

94 // z ! contour levels in increasing order

95

96 #endif

Graphics

API for Graphics windows.

**Definition:** graph.h:36

conrec

int conrec(double \*\*d, int ilb, int iub, int jlb, int jub, double \*x, double \*y, int nc, double \*z, Graphics \*g, int colour=1)

Paul Bourke's conrec algorithm to draw contour lines of PDE fields.

**Definition:** conrec.cpp:57


---

Generated on Thu Aug 14 2014 22:04:01 for Tissue Simulation Toolkit by  

 1.8.6
